# Stability of maxilla after segmental Le Fort I osteotomy combined with anterior maxilla clockwise rotation in patients with maxillary hypoplasia: a retrospective study

**DOI:** 10.1186/s12903-025-06057-4

**Published:** 2025-05-27

**Authors:** Fengqi Song, Xinyu Xu, Zili Li, Xiaojing Liu

**Affiliations:** Department of Oral and Maxillofacial Surgery, Peking University School and Hospital of Stomatology & National Center of Stomatology & National Clinical Research Center for Oral Diseases & National Engineering Research Center of Oral Biomaterials and Digital Medical Devices, No.22, Zhongguancun South Avenue, Haidian District, Beijing, 100081 PR China

**Keywords:** Anterior maxillary clockwise rotation, Segmental Le Fort I osteotomy, Maxillary hypoplasia, Stability

## Abstract

**Background:**

Segmental Le Fort I osteotomy combined with anterior maxillary clockwise rotation has been proposed as an effective treatment for maxillary hypoplasia. However, the stability of maxilla after the operation remains unknown.

**Methods:**

A total of 30 patients undergoing segmental Le Fort I osteotomy were retrospectively included. The follow-up period was more than one year. The stability of anterior maxilla after clockwise rotation was evaluated by cone beam computed tomography (CBCT) performed before surgery (T0), three days after surgery (T1), and at least one year after surgery (T2), respectively. The key parameters were the postoperative relapse of the anterior maxillary clockwise rotation angle (CRA) and paranasal advancement.

**Results:**

Following segmental Le Fort I osteotomy, the average CRA of the anterior maxilla was 10.02° ± 3.86°, while the mean paranasal advancement was 6.22 ± 1.40 mm. At the one-year follow-up, the relapse of CRA and paranasal advancement were **-**0.42° ± 2.51° (*p* = 0.951) and -0.28 ± 0.83 mm (*p* = 0.08), respectively, suggesting good postoperative stability. Additionally, no significant correlation was found between the intraoperative CRA and its relapse over time.

**Conclusion:**

Segmental Le Fort I osteotomy combined with anterior maxillary clockwise rotation demonstrates favorable stability up to one year postoperatively, making it a reliable approach for the treatment of maxillary hypoplasia.

## Background

Skeletal Class III dentofacial deformity, characterized by maxillary hypoplasia and mandibular hyperplasia, presents with both facial disharmony and malocclusion. A common compensatory feature in these patients is the labial inclination of the upper incisors, which can be addressed through preoperative orthodontic treatment. However, this process is often time-consuming and may not always yield optimal results [1, 2]. Furthermore, in patients with Skeletal Class III dentofacial deformities, the labial cortical plate of the upper incisors is typically thin, limiting the extent of orthodontic decompensation and increasing the risk of periodontal complications [3, 4]. Even with premolar extraction and prolonged preoperative orthodontic treatment, achieving an ideal upper incisor angulation remains challenging, and patients may develop periodontal defects in the process [5, 6]. Clockwise rotation of the maxillomandibular complex (MMC) during the orthognathic surgery has been proposed as a strategy to optimize the upper incisor axis. [7]. However, MMC clockwise rotation is constrained by the occlusal plane angle, as excessive steepening occlusal plane may compromise incisal guidance or introduce functional interferences in the posterior dentition, leading to prolonged masticatory discomfort [8, 9].

To overcome these limitations, Dr. Chen and his team introduced a novel surgical approach combining segmental maxillary osteotomy, anterior maxillary clockwise rotation, and bilateral premolar extraction. This method is particularly suit for patients with pronounced labial inclination of the upper incisors [10, 11]. By directly correcting the compensatory inclination surgically, this approach minimizes the need for extensive preoperative orthodontic decompensation while circumventing occlusal plane constraints. However, the anterior maxillary clockwise rotation may increase tension in the palatal mucoperiosteum, potentially affecting postoperative skeletal stability [12–14].

Despite its promising advantages, no studies have yet evaluated the postoperative skeletal stability of this technique. Therefore, this retrospective study aims to assess the stability of the maxilla following segmental Le Fort I osteotomy with anterior maxillary clockwise rotation in patients with Class III dentofacial deformities. Specifically, we seek to determine whether anterior maxillary clockwise rotation provides stable clinical outcomes and whether the degree of rotation is associated with postoperative maxillary stability.

## Methods

### Patients

This retrospective study was approved by the Institutional Review Board of Peking University, School of Stomatology (approval number PKUSSIRB-202278111) and conducted in accordance with the Declaration of Helsinki guidelines for human studies. The inclusion criteria were as follows: (1) patients diagnosed with skeletal Class III dentofacial deformity; (2) patients who underwent anterior maxillary clockwise rotation combined with segmental Le Fort I osteotomy and bilateral premolar extraction; (3) surgical plans formulated based on the computer-assisted surgical simulation (CASS) protocol; and (4) patients aged between 18 and 40 years. The exclusion criteria were as follows: (1) patients with secondary deformities related to cleft lip/palate or facial trauma; (2) patients with systemic diseases; (3) patients with active periodontal disease or severe bone loss and (4) patients with a follow-up period of less than one year.

### Data acquisition

Preoperative virtual planning followed the standard CASS protocol. Data acquisition included cone-beam computer tomography (CBCT) scans (NewTom VGi; Cefla S.C., Verona, Italy) with a 16 cm × 16 cm field of view, stored in Digital Imaging and Communications in Medicine (DICOM) format. Natural head position (NHP) was recorded using a multicamera system (3 dMD, Atlanta, GA) and a laser level (SaiWei, Shanghai, China). A 3D laser surface scanner (3Shape, Copenhagen, Denmark) with a 0.1 mm resolution was used to capture digital morphology of the upper and lower dentitions. DICOM data were imported into the virtual surgical planning software IVSP Image Trial (version 1.0.24.36, IVSPlan, Beijing, China). Skeletal, dental, and textured skin morphologies were superimposed using a surface reconstruction process and aligned with the NHP by adjusting the x, y, and z axes based on laser level markers from the 3D face scan [15].

### Preoperative virtual planning

**Fig 1:**
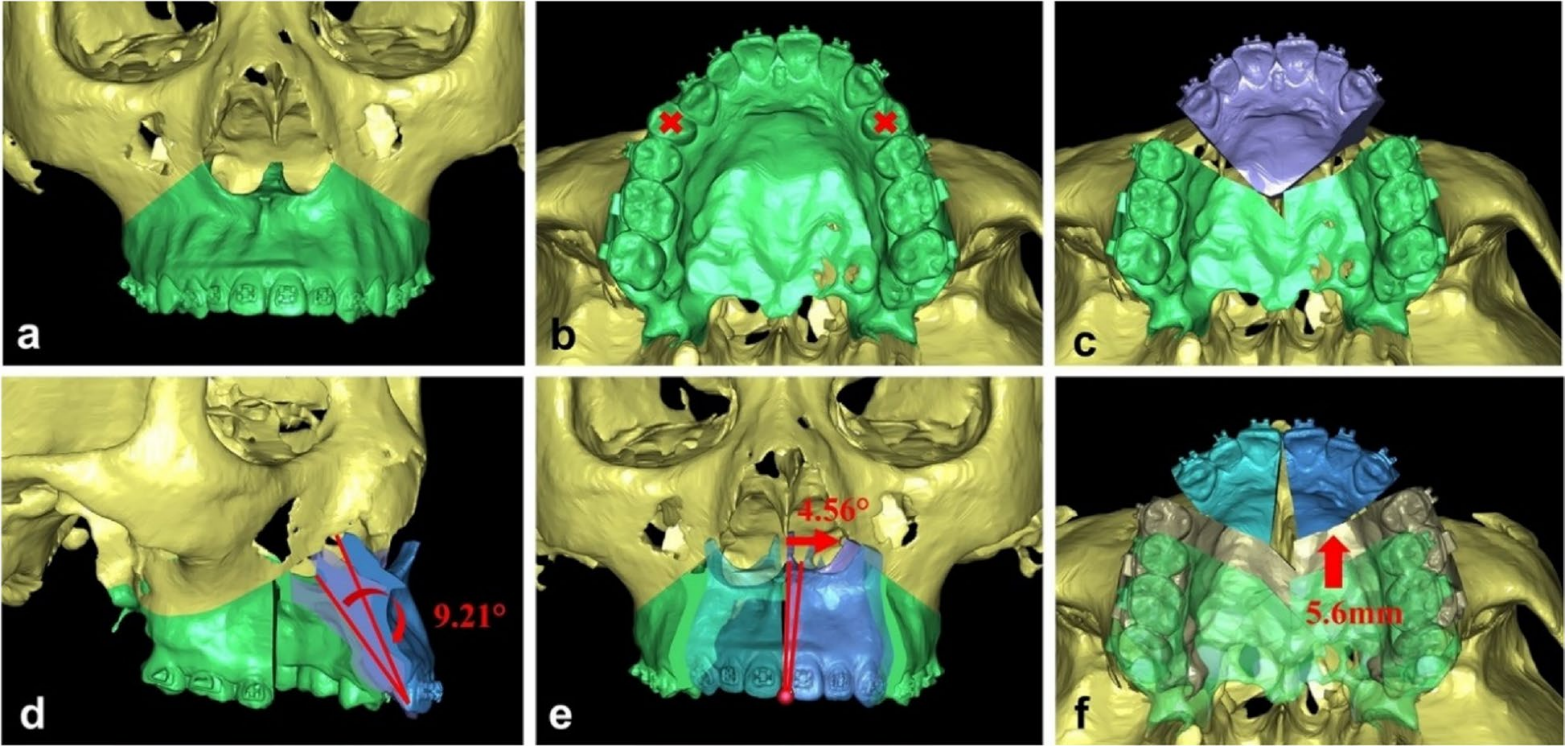
Virtual design of segmental Le Fort I osteotomy. **a** Initial maxilla and the upper dentition. **b** Bilateral first premolars were extracted. **c** The maxilla was divided into the anterior and posterior parts. **d** The anterior maxillary segments were rotated clockwise centering on the upper incisor point. **e** The anterior bone segments were rotated in the coronal plane to decrease the steps between canines and second premolars. **f** The posterior maxilla advanced to close the residual space

The virtual planning process is illustrated in Fig. 1. First, bilateral premolars were virtually extracted (Fig. 1b), followed by segmental Le Fort I osteotomy (Fig. 1c) and bilateral sagittal split ramus osteotomy (BSSRO). The anterior maxilla was then rotated clockwise around the upper incisor point (U1) to achieved the desired upper incisor inclination and maxilla convexity (Fig. 1d). The upper and lower dentitions were re-established virtually to ensure proper canine, molar, and incisor relationships. If clockwise rotation caused bilateral canines to deviate from the occlusal plane, a midline osteotomy was performed in the anterior maxilla. To facilitate occlusal alignment, bilateral anterior bone segments-centered on U1-were rotated oppositely in the coronal plane (Fig. 1e). Residual extraction spaces were closed by anterior movement of the upper molars (Fig. 1f) to optimize occlusion and achieve stable bilateral molars contacts.

The maxillary bone segments and distal mandibular body were aligned according to the new occlusion as the MMC. The MMC was repositioned relative to the skull base to meet aesthetic objectives. Paranasal advancement was assessed in the virtual plan for verification in the OR room. Intermediate and final surgical splints were designed accordingly and fabricated using stereolithography-based 3D printing.

### Surgical procedure

**Fig 2:**
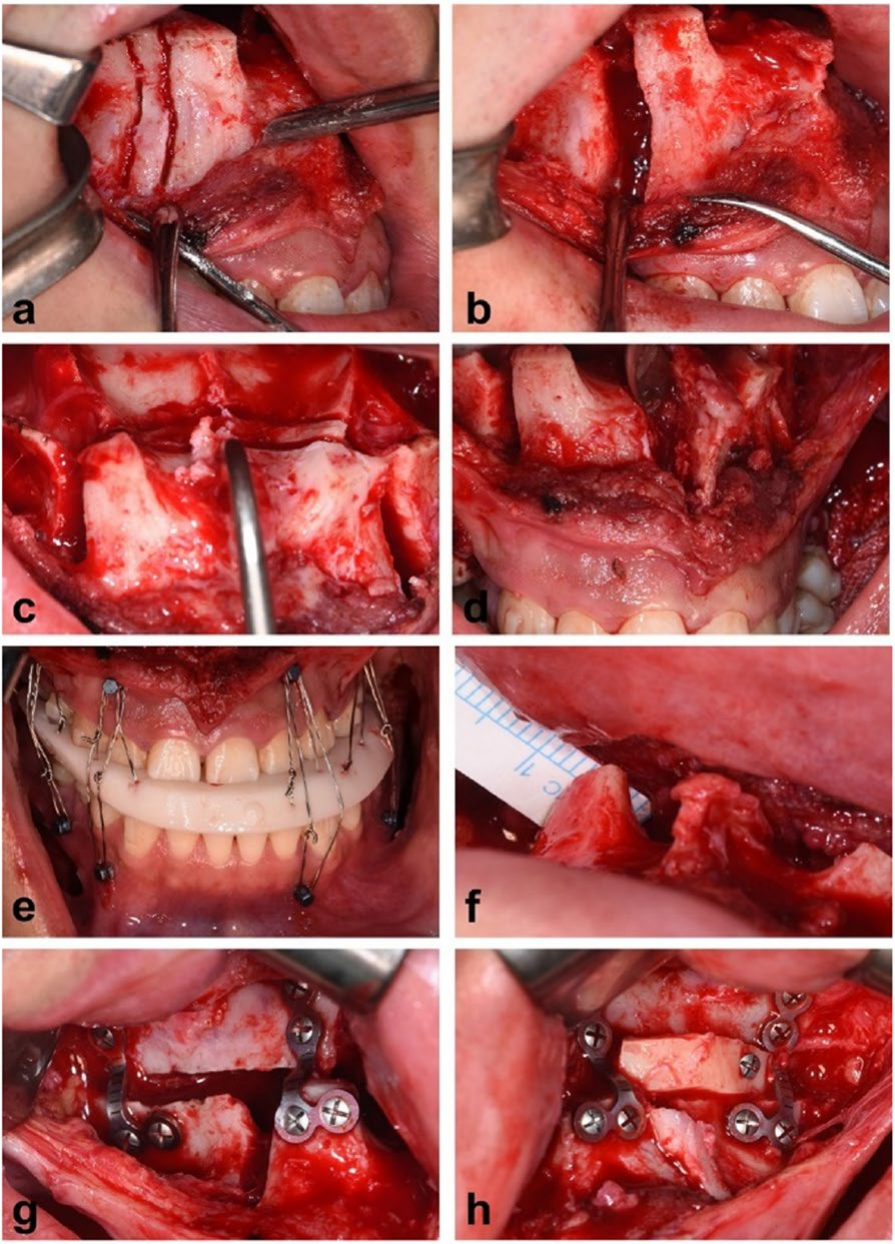
Surgical procedure of segmental Le Fort I osteotomy. **a** Vertical interradicular osteotomies adjacent to the extraction socket. **b** Removal of the intervening bone. **c** Palatal osteotomy lines. **d** Central vertical interradicular osteotomy. **e** Intermaxillary fixation. **f** Measurements and verification of paranasal advancement compared to the preoperative virtual design. **g** Rigid internal fixation. **h** Autologous bone grafting

Surgery was performed under general anesthesia. A vestibular incision was made 5 mm above the attached mucosa, extending from the right to the left first molar region. A mucoperiosteal flap was elevated to expose the anterior maxillary wall, the zygomaticomaxillary buttress, and nasal piriform aperture. Bilateral Le Fort I osteotomy was carried out using a surgical bur and reciprocating saw, beginning at the posterior lateral maxilla, passing through the zygomatic buttress along the anterior maxillary wall, and terminating at the piriform apertures. Bilateral premolars were then extracted, followed by vertical interradicular osteotomies adjacent to the extraction socket (Fig. 2a-b). The vertical osteotomy lines extended across the alveolar ridge and converged horizontally at the middle of the palate (Fig. 2c). The intervening bone was excised accordingly. The anterior maxillary bone segments were repositioned (Fig. 2d), with the upper dentition realigned within the intermediate surgical splint (Fig. 2e). Intraoperative measurements of the paranasal region were carefully compared to the preoperative virtual plan (Fig. 2f). Titanium plates were shaped and fixed to facilitate paranasal advancement (Fig. 2g), and autologous bone grafting was performed to fill any remaining gaps (Fig. 2h). The final splint was retained for six weeks postoperatively before removal. BSSRO with rigid fixation was performed uniformly in all cases.

### Postoperative analysis

Postoperative evaluation was performed using Dolphin 3D 11.95 (Dolphin Imaging & Management Solutions, Chatsworth, CA) and IVSP Image Trial (version 1.0.24.36, IVSPlan, Beijing, China). CBCT scans were obtained at three time points: preoperative (T0), three days postoperatively (T1), and at least one year postoperatively (T2). Images were superimposed using a voxel-based cranial base registration protocol [16] in Dolphin 3D, and the registered DICOM files were imported into IVSP Image Trial for 3D reconstruction and cephalometric analysis. Postoperative skeletal changes were assessed by comparing pre- (T0) and immediate post-operative (T1) parameters. While postoperative stability was analyzed by comparing immediate postoperative (T1) and one-year postoperative (T2) images.

**Fig. 3:**
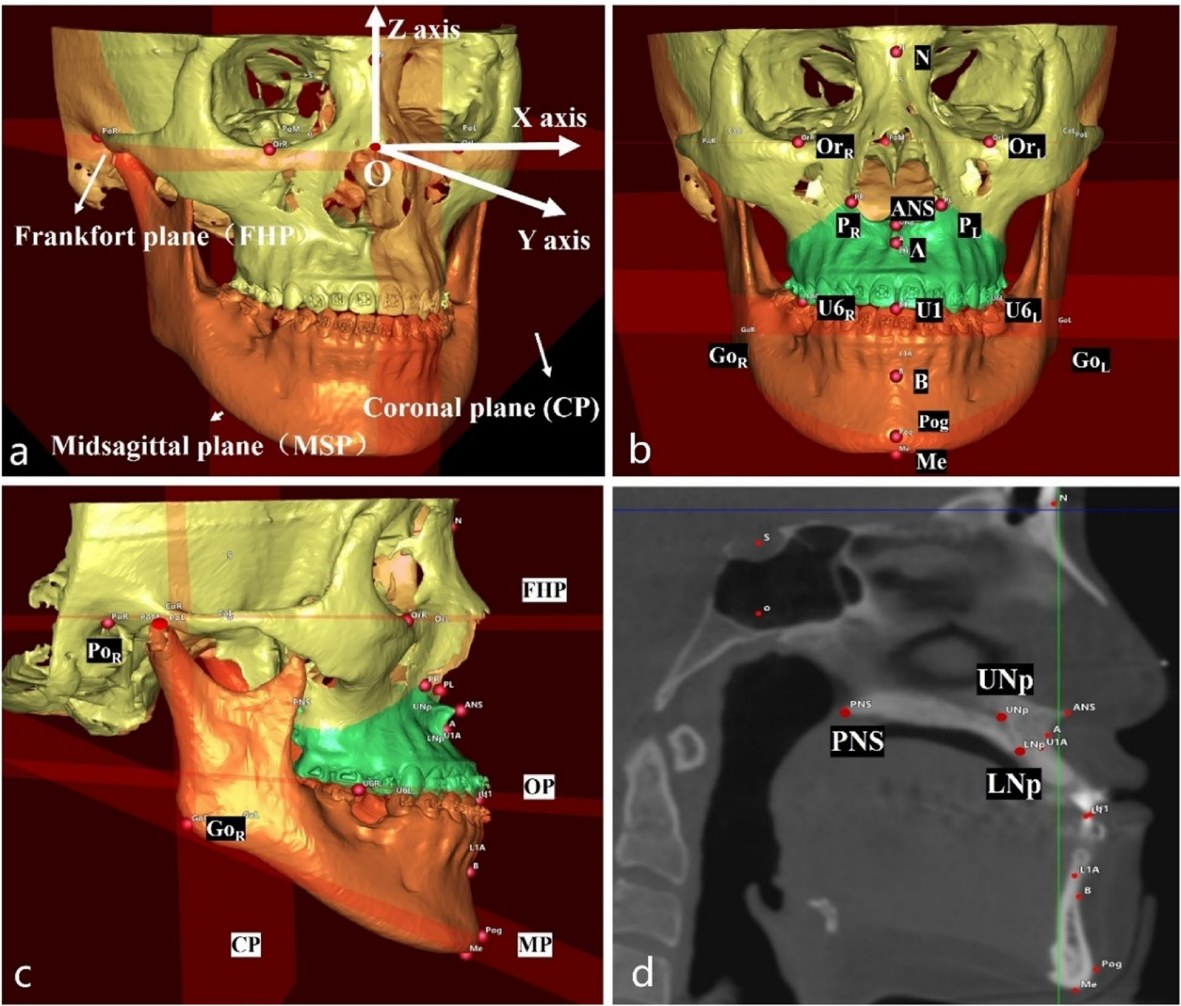
Reference coordinate system and landmarks used for 3D cephalometry. **a** Reference coordinate system adhered to a left-handed orientation. **b** Front view of the twenty landmarks. **c** Sagittal view of the twenty landmarks and the planes. **d** Landmarks selection in DICOM data

A standardized reference coordinate system was established, incorporating the Frankfort plane (FHP), midsagittal plane (MSP), and coronal plane (CP) (Table 1). The coordinate system origin (point O) was defined at the intersection of FHP, MSP, and CP. The X-axis represented the intersection of FHP and CP, Y-axis was the intersection of FHP and MSP, and the Z-axis defined by the intersection of MSP and CP. This coordinate system adhered to a left-handed orientation (Fig. 3a).

**Table 1:** Landmarks and planes formulated in the 3D coordinate system to assess postoperative stability

**Landmarks**	**Abbreviations**	**Definitions**
Sella	S	The center of the hypophyseal.
Nasion	N	The midpoint of the frontonasal suture.
Orbital	Or_L_/Or_R_	The most inferior point of the infraorbital rim.
Porion	Po_L_/Po_R_/Po_M_	The most superior point of the external acoustic meatus. Po_M_ is the midpoint of Po_L_ and Po_R._
Apertura piriformis point	P_L_/P_R_	The most lateral point of the margin of the piriform aperture
Subspinale	A	The point of the maximum concavity in the midline of the dentoalveolar process of the maxilla
Upper incisor	U1	The most mesial point of the tip of the crown of the right upper central incisor.
Upper incisor apex	U1 A	The superior tip of the root of the right upper central incisor
Upper nasopalatine canal point	UNp	The most superior point of the posterior margin of the nasopalatine canal
Lower nasopalatine canal point	LNp	The most inferior point of the posterior margin of the nasopalatine canal
Anterior nasal spine	ANS	The most anterior nasal spine
Posterior nasal spine	PNS	The most posterior midpoint of the posterior nasal spine of the palatine bone
Upper molar point	U6_L_/U6_R_	The most inferior point of the mesial cusp of the crown of the first upper molar in the profile plane
Supramental	B	The point of maximum concavity in the midline of the dentoalveolar process of the mandible
Pogonion	Pog	The most anterior point of the chin
Menton	Me	The most inferior midpoint of the chin on the outline of the mandibular symphysis
Gonion	Go_L_/Go_R_	The point at the mandibular angle, defined by a perpendicular to the intersection point of the tangent lines to the posterior margin of the vertical ramus and the inferior margin of the mandibular body
**Planes**	**Abbreviations**	**Definitions**
Frankfort plane	FHP	The plane crossing OrL, OrR and PoM
Midsagittal plane	MSP	The plane passing S and N and perpendicular to FHP
Coronal plane	CP	The plane passing S and perpendicular to FHP and MSP
Occlusal plane	OP	The plane passing U1, U6_R_ and U6_L_
Mandible plane	MP	The plane passing Go_R_, Go_L_ and Me

Twenty anatomical landmarks were identified within the 3D coordinate system (Fig. 3b-d) to assess maxillary stability (Table 1). Key stability indicators included the clockwise rotation angle (CRA) of the anterior maxilla (CRA_T1-T2_) and the sagittal displacement of the apertura piriformis point (Δ P_T1-T2_). Mandibular stability was primarily evaluated using the sagittal displacement of the supramental point (Δ B_T1-T2_).

The CRA from T0 to T1 (CRA_T0-T1_) was determined by the difference between the upper incisor inclination change (ΔU1-FHP _T0-T1_) and the occlusal plane rotation (ΔOP-FHP _T0-T1_):

1 $${CRA}_{(T0-T1)}=\left|\Delta U1-{FHP}_{\left(T0-T1\right)}\right|-\Delta OP-{FHP}_{(T0-T1)}$$

To minimize postoperative orthodontic influence, CRA_T1-T2_ was calculated using the difference between the anterior maxillary alveolar inclination change (ΔNP-FHP _T1-T2_) and the palatal plane rotation (ΔPP-FHP _T1-T2_):

2 $${CRA}_{(T1-T2)}=-(\Delta NP-{FHP}_{\left(T1-T2\right)}+\Delta PP-{FHP}_{\left(T1-T2\right)})$$

Inter- and intra-examiner reliability was assessed in 10 randomly selected patients. The same examiner (F.S.) marked landmarks twice within one week to evaluated intra-examiner reliability, while two independent examiner (F.S. and X.X.) marked landmarks simultaneously to assess inter-examiner reliability. Both intra- and inter-observer correlation coefficients exceeded 0.95 indicating excellent reproducibility.

### Statistical analysis

Descriptive statistics were presented as means and standard deviations (SD). The distribution of data was assessed using the Shapiro-Wilk test. Normally distributed data were analyzed using paired t-tests, while non-normally distributed data were assessed using the Wilcoxon signed-rank test. Correlation analysis were conducted using Pearson’s correlation for normally distributed data and Spearman’s correlation for non-normally distributed data. Statistical analyses were performed using IBM SPSS Statistics 25.0 (SPSS Inc., Chicago, IL, USA), with *P*-values <0.0 considered statistically significant.

## Results

### Patient demographics

A total of 30 patients met the inclusion criteria and were enrolled in the study (Table 2). Among them, 24 were female (80%) and 6 were male (20%). The mean age at the time of surgery was 24.9 years (range: 18–39 years) and the average follow-up duration was 13.8 months (range: 12–30 months). All patients underwent segmental Le Fort I osteotomy (SLFI) combined with BSSRO, and 18 of them (60%) also underwent genioplasty.

**Table 2:** Patient demographics (*N* = 30)

Variable	Value	Percent
Sex		
Female	24	80%
Male	6	20%
Age (years)		
Mean (range)	24.9	(18–39)
Follow-up (months)		
Mean (range)	13.8	(12–30)
Surgical treatment		
SLFI + BSSRO	30	100%
SLFI + BSSRO + Genioplasty	18	60%
Orthodontic appliances		
Fixed	22	73.3%
Invisible	8	26.7%

*SLFI Segmental Le Fort I osteotomy*

### Maxillary changes after surgery and relapse

Postoperative maxillary changes (T0-T1) and subsequent relapse (T1-T2) are summarized in Table 3. From T0 to T1, the CRA of anterior maxilla and the occlusal plane were 10.02° ± 3.86° and 3.66°± 3.27°, respectively. The labial inclination angle of upper incisors decreased by −13.61 ± 4.05°. The average sagittal backward movement of the upper incisors was −0.63 ± 2.16 mm. Additionally, the paranasal region showed a forward displacement of 6.22 ± 1.40 mm, and the posterior maxilla advanced by 4.16 ± 1.57 mm.

**Table 3:** Postoperative changes and relapse of the maxilla

Measurement	Changes (T0–T1)		Relapse (T1–T2)		
	mean	SD	mean	SD	*p*-value
SNA(°)	5.53	2.78	−0.35	1.38	0.179
CRA(°)	10.02	3.86	−0.42	2.51	−0.915
OP-FHP(°)	3.66	3.27	−2.59	2.54	<0.001^***^
U1-FHP(°)	−13.61	4.05	4.25	7.66	0.005^**^
PP-FHP(°)	3.59	3.31	0.24	1.09	0.239
NP-FHP(°)	−12.98	4.12	0.18	2.06	0.635
U1-NP(°)	−0.05	0.70	4.12	6.99	0.003^***^
A_Y(mm)	3.25	1.42	−0.15	0.79	0.296
A_Z(mm)	1.96	1.32	−0.32	0.58	0.176
P_Y(mm)	6.22	1.40	−0.28	0.83	0.080
U1_Y(mm)	−0.63	2.16	0.63	2.07	0.016^*^
U1_Z(mm)	1.97	1.29	−0.79	1.83	0.024^*^
PNS_Y(mm)	4.16	1.57	0.08	0.63	0.511

One-sample t-test and Wilcoxon signed-rank test, test value = 0

(_Y) sign and (_Z) sign indicate movements in relation to coronal and horizontal planes

(-) sign of measurements of distance indicates movements in the upward and posterior directions in relation to horizontal and coronal planes, respectively

* *P*<0.05 ** *P*<0.01 *** *P*<0.001

During the T1–T2 phase, the CRA of the anterior maxilla showed a minor relapse of −0.42° ± 2.51° (*p* = 0.951, not significant), while the occlusal plane rotated back by −2.59° ± 2.54° (*p* < 0.001). The upper incisors exhibited a forward movement of 0.63 ± 2.07 mm (*p* = 0.016) and a vertical upward movement of −0.79 ± 1.83 mm (*p* = 0.024). The sagittal relapse of the paranasal region was −0.28 ± 0.83 mm (*p* = 0.08, not significant). Additionally, no significant relapse was observed in PP-FHP, NP-FHP, A_Y, A_Z, or PNS_Y.

### Mandibular changes after surgery and relapse

Postoperative mandibular changes (T0–T1) and subsequent relapse (T1–T2) are presented in Table 4. From T0 to T1, the B point moved posteriorly by −6.51 ± 2.56 mm and vertically downward by 3.28 ± 1.70 mm. The SNB angle decreased by −3.91° ± 1.98°, while the FMA increased by 2.06° ± 3.53°.

**Table 4:** Postoperative changes and relapse of the mandible

Measurement	Changes (T0–T1)		Relapse (T1–T2)		
	mean	SD	mean	SD	*p*-value
SNB(°)	−3.91	1.98	1.79	1.28	<0.001^***^
FMA(°)	2.06	3.53	−2.31	2.49	<0.001^***^
B_Y(mm)	−6.51	2.56	1.71	1.94	<0.001^***^
B_Z(mm)	3.28	1.70	2.12	1.99	<0.001^***^
Pog_Y(mm)	−6.05	4.27	2.21	2.20	<0.001^***^

One-sample t-test and Wilcoxon signed-rank test, test value = 0

(_Y) sign and (_Z) sign indicate movements in relation to coronal and horizontal planes

(-) sign of measurements of distance indicates movements in the upward and posterior directions in relation to horizontal and coronal planes, respectively

* *P*<0.05 ** *P*<0.01 *** *P*<0.001

During T1–T2, the B point exhibited a forward and upward relapse of 1.71 ± 1.94 mm (*p *< 0.001) and 2.12 ± 1.99 mm (*p* < 0.001), respectively. SNB increased by 1.79° ± 1.28° (*p* < 0.001), and FMA decreased by −2.31° ± 2.49° (*p* < 0.001).

### Correlation analysis of variations after surgery and at relapse

**Fig 4:**
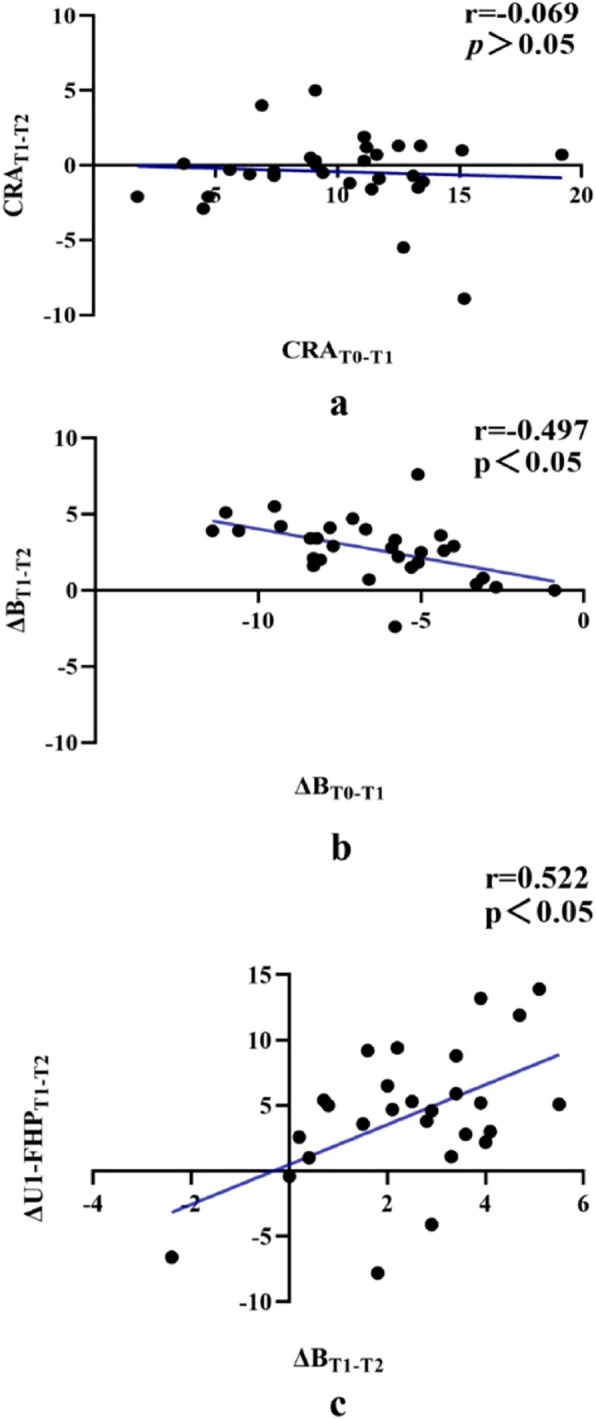
Correlation analysis results. **a** Pearson’s correlation between CRA_T1-T2_ and CRA_T0-T1_. **b** Pearson’s correlation between ΔB_T1-T2_ and ΔB_T0-T1_. **c** Pearson’s correlation between of ΔB_T1-T2_ and ΔU1-FHP_T1-T2_. *P* <0.05 was considered statistically significant

Correlation analysis revealed that the CRA of the anterior maxilla after surgery (CRA_T0-T1_) had no significant correlation with its relapse (CRA_T1-T2_) (Fig. 4a). However, mandibular relapse during the initial 12-month follow-up (ΔB_T1-T2_) was positively correlated with the magnitude of mandibular setback after surgery (ΔB_T0-T1_). (Fig. 4b). Furthermore, mandibular relapse (ΔB_T1-T2_) exhibited a positive correlation with the postoperative change in the labial inclination angle of the upper incisors (∆U1-FHP_T1-T2_) (Fig. 4c).

## Discussion

Segmental Le Fort I osteotomy is a versatile surgical technique for addressing complex maxillary deformities [17]. The postoperative stability of the maxilla is influenced by multiple factors, including the osteotomy method, the number of segments, the magnitude and direction of segmental movement, bone quality, internal fixation techniques, and bone grafting [18]. This study specifically investigated the effects of segmental movement magnitude and direction on the stability of the anterior maxilla following clockwise rotation. To minimize confounding variables, other factors, such as the osteotomy method, number of segments, internal fixation techniques, and bone grafting, were standardized across cases. Additionally, patients with paper-thin maxilla were excluded to avoid potential bias in outcomes.

Previous research assessing maxillary stability after segmental osteotomy have primarily relied on two-dimensional imaging and have rarely accounted for variations in osteotomy techniques, limiting their ability to control confounding factors and generalize findings [19–21]. With advancement of 3D imaging technology, CBCT becomes indispensable for analyzing bone segment repositioning after osteotomy [22, 23]. Furthermore, 3D imaging allows for a more precise assessment of postoperative segmental stability [23].In this study, CBCT was used to evaluate the stability of anterior maxilla after clockwise rotation and to explore whether the degree of relapse was associated with the initial rotation angle. Although CBCT provides more imaging information, it also has certain errors, such as image registration errors and anatomical landmark selection errors. To minimize these errors, all CBCT scans were acquired using a standardized protocol, and voxel-based superimposition techniques were applied to ensure accurate alignment across different time points. Additionally, intra- and inter-observer reliability tests were conducted, demonstrating high reproducibility (correlation coefficients >0.95).

Due to tension of the palatal mucoperiosteum, intraoperative maxillary expansion is considered to increases the risk of postoperative relapse [12, 13]. A finite element model study by Sommerfeld et al. demonstrated that palatal mucoperiosteum tension increases proportionally with expansion, reaching 6‒8 MPa when the posterior maxilla is expanded by approximately 5 mm [14]. In our study, clockwise rotation of the anterior maxilla may have encountered a similar challenge. To preserve adequate blood supply, excessive detachment of the mucoperiosteum should be avoided during surgery [2]. Therefore, as the rotation angle increases, the resulting tension in the palatal mucoperiosteum may contribute to postoperative relapse.

In this study, the average intraoperative CRA of the anterior maxilla was 10.02° ± 3.86°. with a mean relapse of −0.42° ± 2.51° one year postoperatively. The inclination angle of the upper anterior alveolar bone and the sagittal position of the paranasal landmarks (PR/PL) retained stable compared to immediate postoperative measurements. These findings suggest that within a rotation range of approximate 10°, the anterior maxilla could maintain satisfactory postoperative stability. Furthermore, correlation analysis revealed no significant association between postoperative relapse and the intraoperative CRA, supporting the reliability of clockwise rotation.

Since the spaces created by premolar extraction were partially closed by posterior maxillary advancement, we also evaluated the stability of posterior maxilla. The mean advancement of the PNS immediately after surgery was 4.16 ± 1.57 mm, with a minimal sagittal change of 0.08 ± 0.63 mm at 12 months postoperatively, indicating good stability of posterior maxilla.

In contrast, the stability of the mandible was inferior to that of the maxilla one year postoperatively. The mean increase in SNB was 1.79 ± 1.28°, and Point B exhibited both anterior and superior displacement, indicating a counterclockwise rotation trend of the mandible. Correlation analysis demonstrated that greater mandibular setback was associated with higher relapse, consistent with previous studies showing that skeletal relapse increases with the extent of mandibular setback in single-jaw surgeries [24, 25]. Findings from studies on double-jaw surgery also indicate that mandibular relapse rates are relatively higher than maxillary relapse rates [26, 27]. Several factors contribute to relapse at point B, including dental relapse, forceful posterior condylar positioning, anterior mandibular rotation following surgical splint removal, and impingement of the pterygomasseteric sling [25, 28–30]. Additionally, as the mandible progressively protrudes postoperatively, the upper incisors undergo labial inclination to maintain occlusion with the lower incisor [11, 31].

However, in segmental Le Fort I osteotomy cases, mandibular protrusion induced labial inclination is more complex, as both the upper incisors and the anterior maxillary segment are subject to movement. Therefore, when evaluating skeletal stability, it is essential to distinguish between changes caused by upper incisor inclination and those resulting from anterior maxillary segment movement. Since traditional two-dimensional radiographs cannot effectively separate dental and alveolar inclination, we used CBCT in this study. The results demonstrated a significant increase in the upper incisor labial inclination 12 months postoperatively, which correlated positively with mandibular relapse. Meanwhile, the changes in NP-FHP and U1-FHP from T1 to T2 were 0.18 ± 2.06° and 4.25 ± 7.66°, respectively, indicating that while the upper incisors exhibited significant labial inclination, the anterior alveolar bone remained stable. Accurately differentiating between skeletal and dental changes allows surgeons to identify factors causing postoperative relapse and implement appropriate interventions. Previous studies have reported that bone anchorage techniques can effectively control the labial inclination of the upper incisors, thereby reducing mandibular relapse [32, 33].

This study primarily aimed to optimize the anterior maxillary rotation angle to enhance the aesthetic outcome of paranasal concavity correction. The advancement of the paranasal landmarks in our study exceeded that reported in previous studies using MMC clockwise rotation [34–36]. Compared to MMC clockwise rotation or advancement of the whole maxilla, this approach offers greater flexibility, as it is not constrained by factors such as the sagittal position of the upper incisors, mandibular plane angle, or occlusal plane angle. However, further studies are needed to comprehensively evaluate the aesthetic benefits of this approach.

This study has certain limitations. The postoperative follow-up period was limited to 12 months, which only permits short-term assessment of maxillary stability following orthognathic surgery. While some studies suggest that most skeletal relapse occurs within six months after surgery [26], long-term observation is necessary to assess soft and hard tissue remodeling. Additionally, due to sample size limitations, only a preliminary analysis of relapse-related factors was conducted. A more in-depth analysis requires a larger sample size and stricter control of confounding variables. Furthermore, this study did not provide information on functional outcomes, such as changes in bite function, speech, or patient-reported satisfaction. Future studies incorporating patient-reported outcomes could provide valuable additional insights.

As a retrospective study, it has inherent limitations, including reliance on pre-existing data, which may lead to incomplete or inconsistent information, as well as the potential for selection and recall bias. Despite these limitations, our study provides meaningful insights into the stability of the maxilla following segmental Le Fort I osteotomy combined with anterior maxillary clockwise rotation in patients with maxillary hypoplasia. These findings may serve as a valuable reference for future prospective studies.

## Conclusions

The combination of segmental Le Fort I osteotomy and anterior maxillary clockwise rotation provides a stable maxillary structure up to one year postoperatively and is clinically feasible for patients with maxillary hypoplaisa. Within a certain range of rotation angles, this technique does not increase the risk of postoperative relapse of the anterior maxilla. However, a tendency for mandibular protrusion was observed postoperatively, accompanied by labial inclination of the upper incisors relative to the alveolar bone. This finding highlights the need for careful postoperative management to minimize potential occlusal and aesthetic implications.

## Data Availability

The datasets used and/or analysed during the current study are available from the corresponding author on reasonable request.

## Abbreviations

MMC: Maxillomandibular complex
CRA: Clockwise rotation of anterior maxilla
BSSRO: Bilateral sagittal split ramus osteotomy
3D: 3-dimensional
